# Supplementary material for: How Actin Polymerization and Myosin II Activity Regulate Focal Adhesion Dynamics in Motile Cells
Source: Int J Mol Sci. 2025 Aug 9;26(16):7701. doi: 10.3390/ijms26167701 (PMC12386929; doi:10.3390/ijms26167701)
Supplement: Supplementary file 1 [file ijms-26-07701-s001.zip › ijms-3608148-Supplementary.pdf]

International Journal of *Molecular Sciences*

MDPI

Article

How Actin Polymerization and Myosin II Activity Regulate Focal Adhesion Dynamics in Motile Cells

Anastasiia Kovaleva [1], Evgeniya Solomatina [1,2], Madina Tlegenova [3], Aleena Saidova [3] and Ivan A. Vorobjev [3,4,*]

Supplementary data

Supplementary Table S1. FA area and brightness after 45 μM blebbistatin treatment measured in fixed specimens and life time-lapse frames of vinculin-RFP expressing cells. The data is shown as median with range.

	FA area, μm2							
	Control		30 min		180 min		360 min	
Cell line	fixed	live	fixed	live	fixed	live	fixed	live
3T3	0.44 (0.09–2.13, n=100)	0.86 (0.08–5.45, n=345)	0.16 (0.04–0.61, n=100)	0.31 (0.08–1.3, n=215)	0.13 (0.04–0.45, n=100)	0.22 (0.05–1.23, n=215)	0.06 (0.09–0.28, n=100)	N/A
U2OS	0.56 (0.12–2.93, n=139)	0.76 (0.09–8.48, n=1396)	0.16 (0.03–1.12, n=201)	0.43 (0.07–2.86, n=307)	0.09 (0.02–0.59, n=229)	0.25 (0.09–1.11, n=121)	0.11 (0.02–0.76, n=209)	N/A
	FA brightness, a.u.							
	Control		30 min		180 min		360 min	
	fixed	live	fixed	live	fixed	live	fixed	live
3T3	4849 (203–24136, n=100)	2823 (276–61131, n=345)	2279 (253–11619, n=100)	1311 (241–10335, n=215)	1392 (200–10404, n=100)	883 (120–7129, n=215)	1309 (44–7374, n=100)	N/A
U2OS	18318 (2357–235444, n=139)	2510 (111–123682, n=1396)	3624 (202–97866, n=201)	870 (127– 4425, n=307)	1424 (131–12785, n=229)	685 (88–3514, n=121)	1640 (17–15151, n=209)	N/A

 https://doi.org/10.3390/ijms26167701

Supplementary Table S2. FA area and brightness after 10 µM Y-27632 treatment measured in fixed specimens and life time-lapse
frames of vinculin-RFP expressing cells. The data is shown as median with range.

	FA area, µm2							
	Control		30 min		180 min		360 min	
Cell line	fixed	live	fixed	live	fixed	live	fixed	live
3T3	0.44 (0.09–2.13, n=100)	0.86 (0.08–5.45, n=345)	0.17 (0.04–0.51, n=100)	0.22 (0.05–2.5, n=207)	0.12 (0.04–0.29, n=100)	0.14 (0.05–0.65, n=218)	0.1 (0.04–0.45, n=100)	N/A
U2OS	0.56 (0.12–2.93, n=139)	0.78 (0.09–8.48, n=1396)	0.21 (0.05–1.1, n=217)	0.47 (0.13–4.37, n=369)	0.13 (0.03–1.2, n=189)	0.34 (0.07–1.27, n=219)	0.13 (0.03–0.37, n=205)	N/A
	FA brightness, a.u.							
	Control		30 min		180 min		360 min	
	fixed	live	fixed	live	fixed	live	fixed	live
3T3	4849 (203–24136, n=100)	2823 (276–61131, n=345)	1418 (306–9419,n=100)	665 (84–7055, n=207)	909.5 (130–5150, n=100)	380 (57–1998, n=218)	1047 (184–6050, n=100)	N/A
U2OS	18318 (2357–235444, n=139)	2510 (111– 123682, n=1396)	5371 (779–34047, n=217)	1049 (127–9877, n=369)	2678 (384–43261, n=189)	684 (102–2650, n=219)	3192 (225–11901, n=209)	N/A

Supplementary Table S3. FA area and brightness after 10 µM ML-7 treatment measured in fixed specimens and life time-lapse frames of vinculin-RFP expressing cells. The data is shown as median with range.

	FA area, µm2							
	Control		30 min		180 min		360 min	
Cell line	fixed	live	fixed	live	fixed	live	fixed	live
3T3	0.44 (0.09–2.13, n=100)	0.86 (0.08–5.45, n=345)	0.29 (0.08–1.34, n=100)	0.15 (0.05–0.66, n=212)	0.18 (0.04–0.71, n=100)	0.12 (0.03–0.54, n=211)	0.19 (0.05–0.85, n=100)	N/A

U2OS	0.56 (0.12–2.93, n=139)	0.78 (0.09–8.48, n=1396)	0.43 (0.93–2.64, n=203)	1.1 (0.2–4.92, n=259)	0.18 (0.05–1.45, n=200)	0.5 (0.08–1.81, n=95)	0.21 (0.45–1.02, n=206)	N/A
	FA brightness, a.u.							
	Control		30 min		180 min		360 min	
	fixed	live	fixed	live	fixed	live	fixed	live
3T3	4849 (203–24136, n=100)	2823 (276–61131, n=345)	2652 (303–24189, n=100)	694.5 (94–5033, n=212)	1540 (256–11930, n=100)	447 (100–4090, n=211)	1884 (290–8190, n=100)	N/A
U2OS	18318 (2357– 235444, n=139)	2510 (111– 123682, n=1396)	14546 (1334–143424, n=203)	3177 (52–57308, n=259)	6839 (853–88706, n=200)	2638 (356– 17266, n=95)	8656 (990–60865, n=206)	N/A

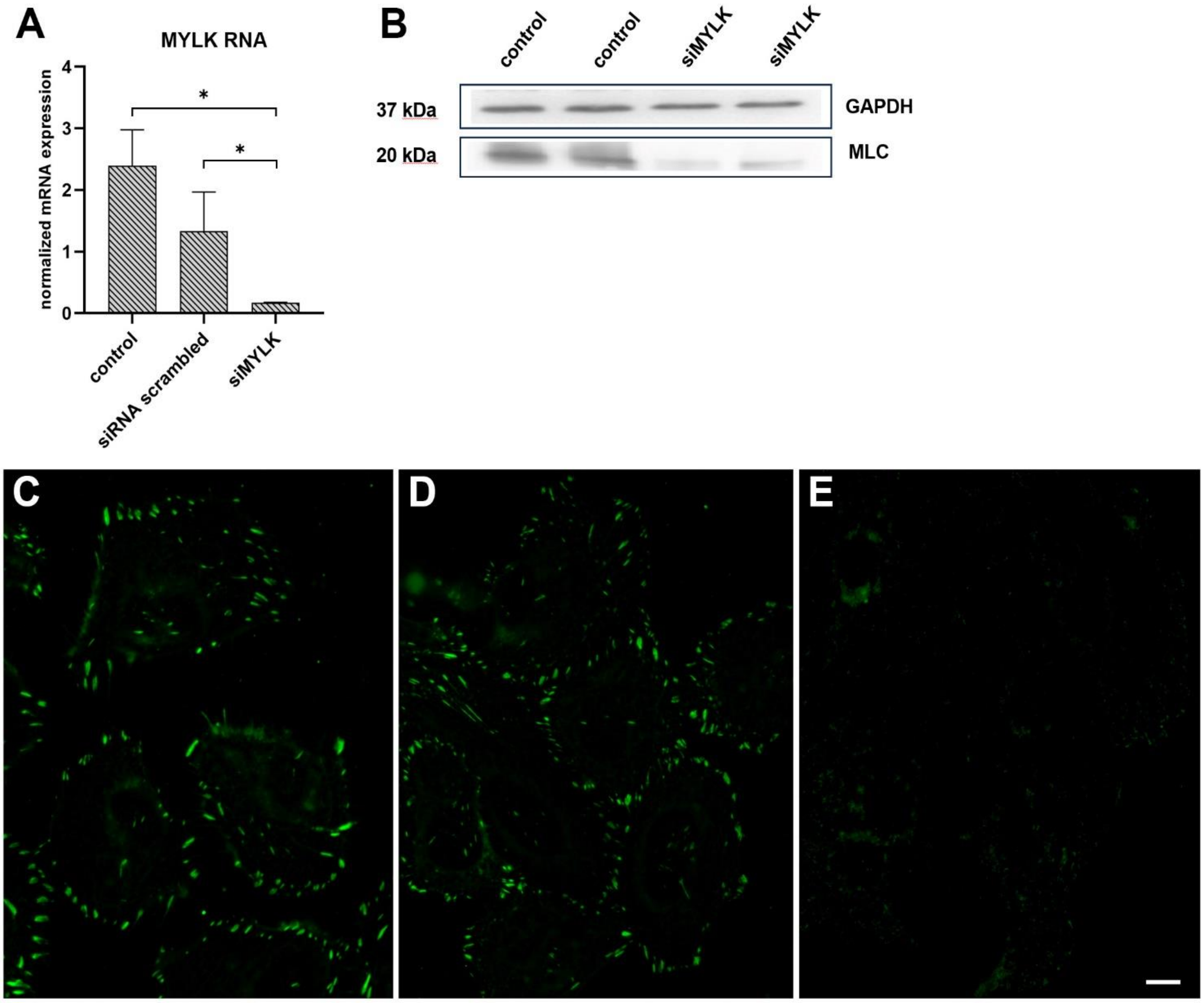

Figure S1. *Reduced expression of MLC at both mRNA and protein levels after MYLK knockdown. (A) RT-qPCR reveals significant reduction of MYLK mRNA in siMYLK-treated cells relative to control and siRNA-scrambled groups, normalized to GAPDH mRNA (*p < 0.05, Mann-Whitney test). (B) Western blot analysis shows decreased MLC protein expression in siMYLK-treated cells compared to control, with GAPDH used for protein normalization. C - FAs in control U2OS cells; D – FAs in mock siRNA treated U2OS cells; E – FAs in siMYLK treated u2OS cells. Scale bar – 10 μm (magnification is the same for three pictures).*

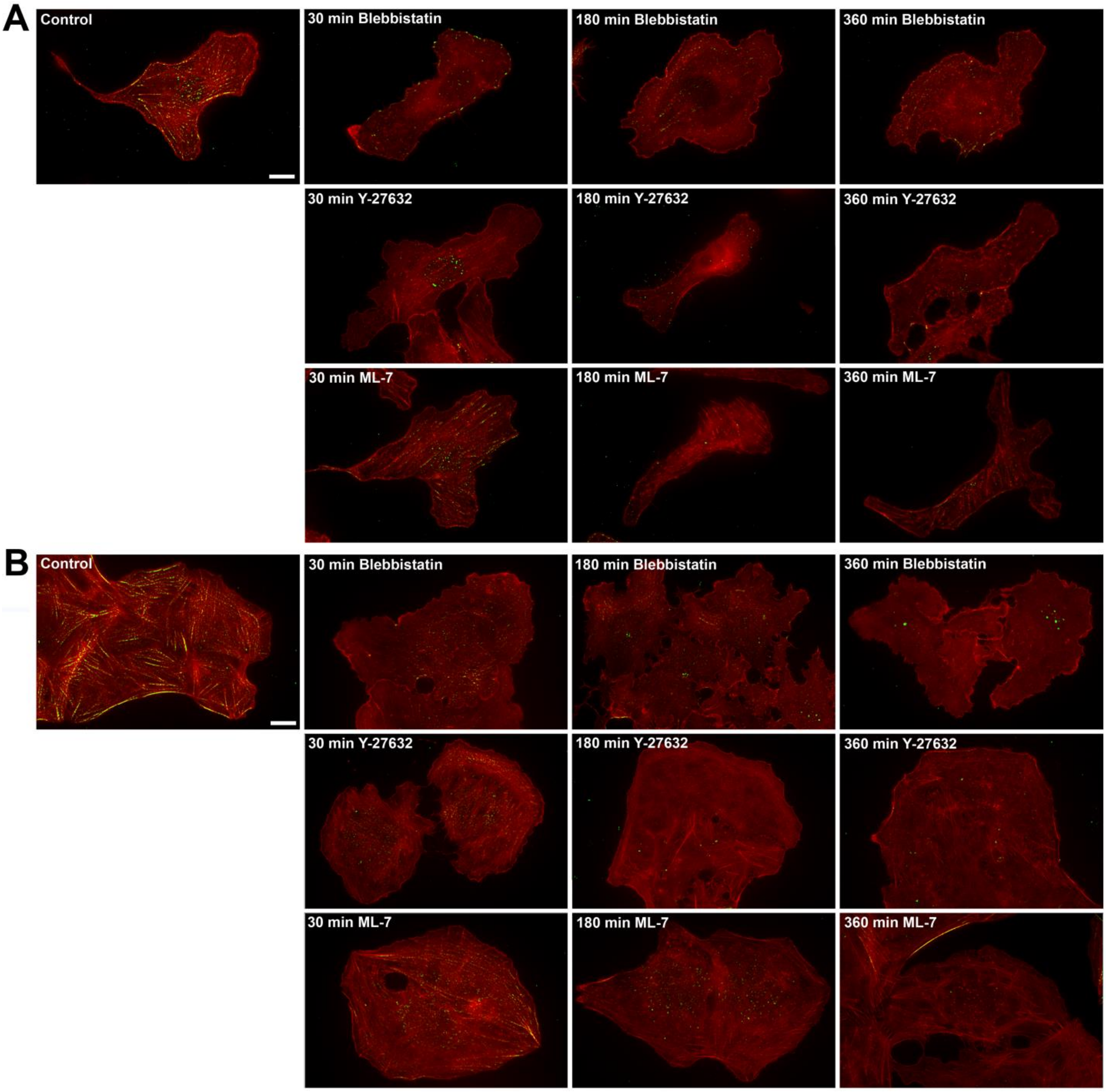

Figure S2. *Actin (red, phalloidin staining) and phosphomyosin II light chains (green, anti-myosin II antibody staining) in 3T3 and U2OS cells under the action of inhibitors of myosin II phosphorylation. Scale bar – 10 μm.*

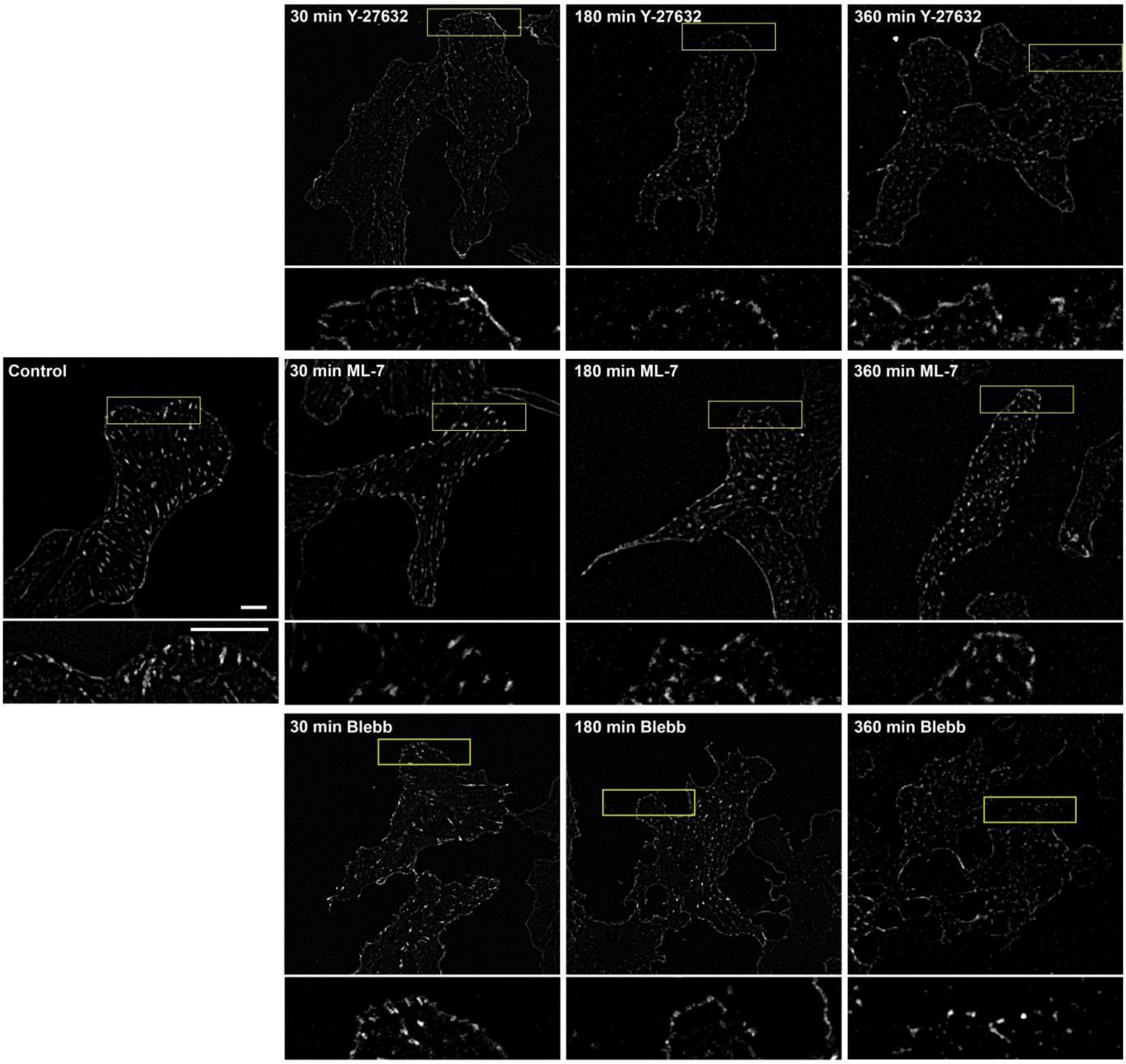

***Figure S3**. FAs in 3T3 vinculin-RFP expressing cell line under the action of myosin II phosphorylation inhibitors. Live cell imaging. FAs are contrasted by difference of Gaussians. In the insets (3x magnification of the highlighted area). Scale bars – 10 µm (magnifications are the same for all upper and lower pictures).*

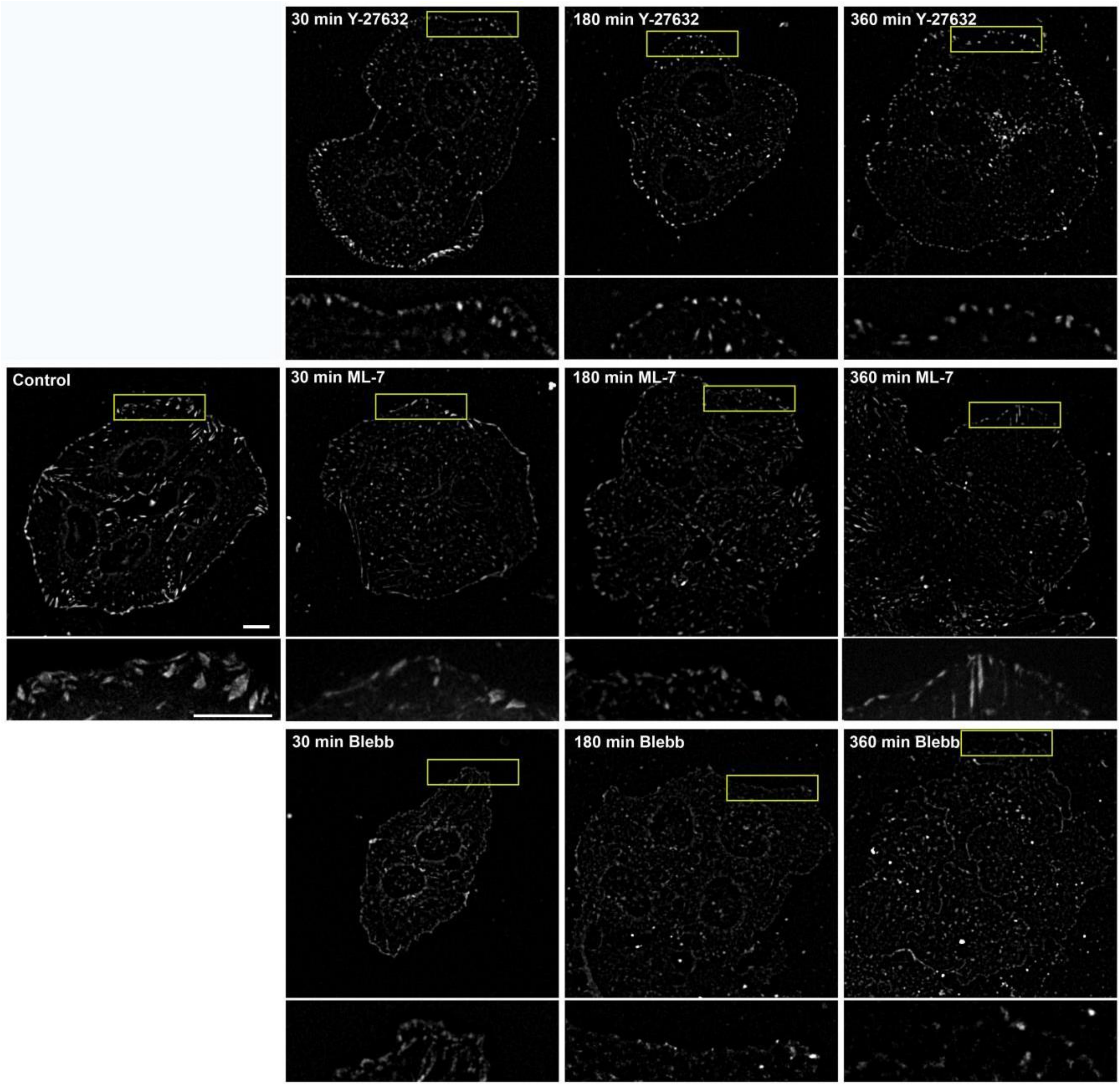

Figure S4. *FAs in U2OS vinculin-RFP expressing cell line under the action of myosin II phosphorylation inhibitors. Live cell imaging. FAs are contrasted by difference of Gaussians. In the insets (3x magnification of the highlighted area). Scale bars – 10 μm*

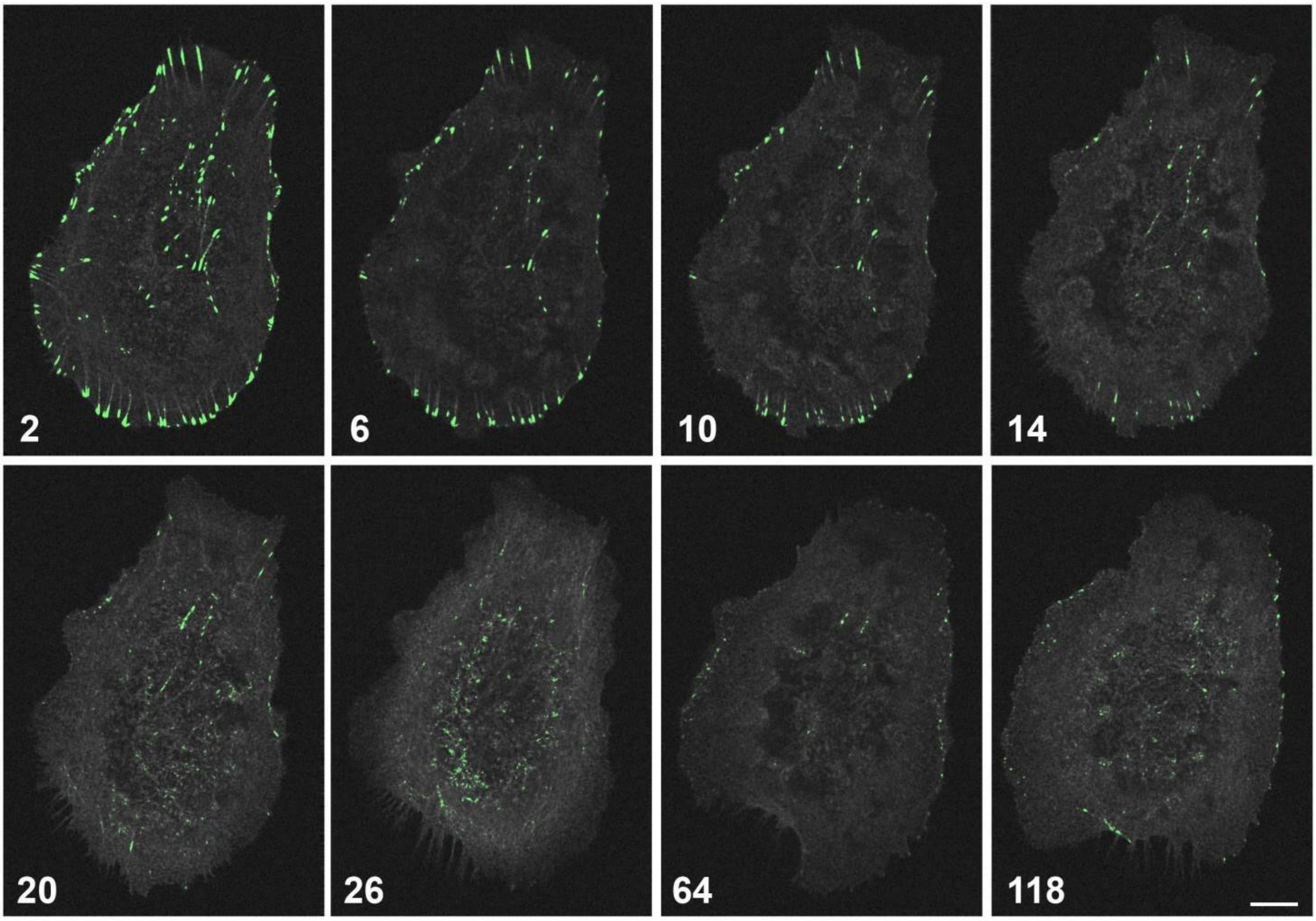

Figure S5. *Dynamics of FAs in vinculin-RFP expressing A549 cells under the action of 45 mM of blebbistatin. Live cell imaging. Notice formation of small FAs at the cell margin after 1 h incubation with the inhibitor. Time after addition of inhibitor is indicated in minutes. FAs are contrasted and highlighted in green. Scale bar – 10 μm.*

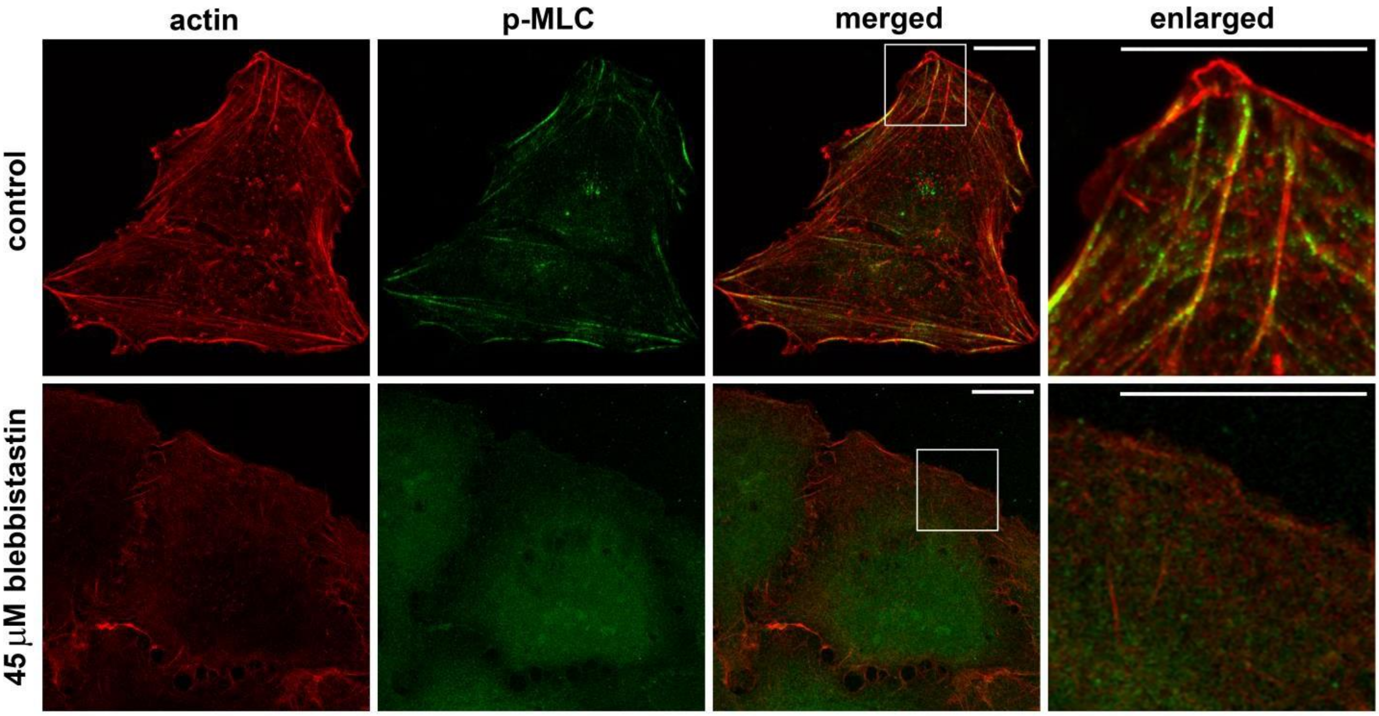

Figure S6. *Actin and phosphorylated myosin in 3T3 cells after the treatment with 45 μM blebbistatin. A – F-actin (phalloidin staining, red) and p-MLC (antibody staining, green). Notice the absence of phosphorylated myosin II near the cell edge after blebbistatin treatment. Scale bars – 10 μm.*

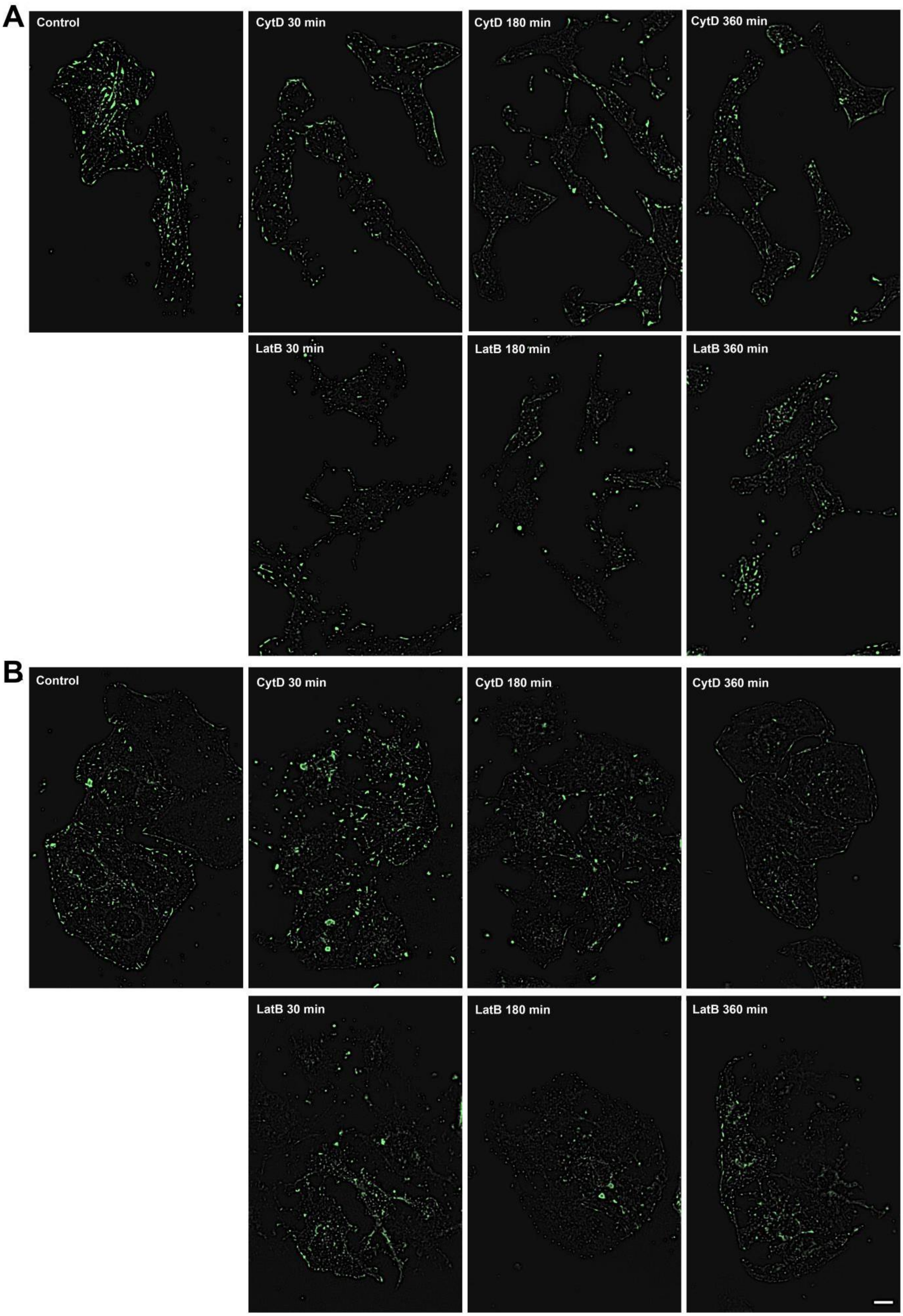
A
Control
CytD 30 min
CytD 180 min
CytD 360 min
LatB 30 min
LatB 180 min
LatB 360 min
B
Control
CytD 30 min
CytD 180 min
CytD 360 min
LatB 30 min
LatB 180 min
LatB 360 min

Figure S7. *FAs in 3T3 (A) and U2OS (B) cells expressing vinculin-RFP under the action of actin polymerization inhibitors (Cyt D – cytochalasin D, 1 µM; LatB – latrunculin B, 1 µM). Duration of treatment is indicated in the upper left corner. FAs are contrasted and highlighted in green. Scale bar – 10 µm.*
